# Sustainability of a field epidemiology and laboratory training programme: the Ghanaian story

**DOI:** 10.11604/pamj.2019.33.68.16431

**Published:** 2019-05-29

**Authors:** Delia Akosua Bandoh, Ernest Kenu, Donne Kofi Ameme, Samuel Oko Sackey, Fredrick Wurapa, Edwin Andrew Afari

**Affiliations:** 1 Ghana Field Epidemiology and Laboratory Training Programme, Department of Epidemiology and Disease Control, School of Public Health, University of Ghana, Legon, Accra, Ghana

**Keywords:** Sustainability, Ghana FELTP, Ghana Health Service

## Abstract

**Introduction:**

Field Epidemiology Training Programmes (FETPs) are functional ways of strengthening epidemiology, surveillance and outbreak response capacity in countries. However, sustainability of FETPs is a major challenge facing most countries especially in Africa. The Ghana Field Epidemiology and Laboratory Training Program (GFELTP) started in 2007 in the University of Ghana School of Public Health as a solution to gaps in the public health workforce. This paper assessed the sustainability strategies embedded in the Ghana Field Epidemiology and Laboratory Training Programme.

**Methods:**

We assessed the sustainability of GFELTP by document reviews and interviews with programme staff and stakeholders to identify sustainability structures (programme, financial and institutional) that were in place. We grouped information into the following headings: programme structure, institutional, financial and political structures.

**Results:**

As of July 2017, a total of 350 public health experts have been trained in both frontline and advanced courses since the programme's inception. For funding structures, the programme is funded mainly by its partners and stakeholders who are local government organisations. They provide resources for running of programme activities. Under institutional and political structures, the programme was established as a Ministry of Health/Ghana Health Service programme based in the University of Ghana. The programme steering committee which is currently chaired by the Director Public Health of Ghana Health Service, jointly ensures its implementation. Other structures of sustainability observed were involvement of stakeholders and alumni in human resource of the programme; use of stakeholders as faculty for the programme. These stakeholders include staff from University of Ghana School of Public Health, Ghana Health Service and Veterinary Service Department, World Health Organization and Centers for Disease Control and Prevention. The programme showed evidence of stable sustainability strategies in all four structures evaluated.

**Conclusion:**

The assessment found the GFELTP to be sustainable. The main factors that contributed to rendering it sustainable were funding, programme, institutional and political structures embedded in the programme. One remarkable sustainability element observed was the strong collaboration that existed between stakeholders of the programme who worked hand in hand to ensure the programme runs smoothly. However, more sources of funding and other essential resources need to be considered to help the programme obtain a pool of resources for carrying out its activities.

## Introduction

Sustainability of public health programmes is of much importance since it helps in maintaining and improving existing health system structures. Field Epidemiology Training Programmes (FETPs) are described by Schneider and colleagues as functional ways of strengthening epidemiology, surveillance and outbreak response capacity in countries [1]. The main aim of FETPs is to build a public health system which can rapidly respond to health threats as they assist in developing scientific evidence-based policies [2]. As an essential public health strategy, existence and functionality of FETPs are key to solving the dynamic public health challenges that keep arising. Thus, there is the need to sustain FETPs to enable them meet their goals. FETPs in Africa were established to strengthen the public health workforce to implement and lead multi-disease surveillance and response in Africa [3]. Sustaining these programmes in Africa in the face of dwindling donor support is therefore of much essence to FETPs in Africa and countries at large [4, 5]. The Ghana Field Epidemiology and Laboratory Training Program (GFELTP) is the Ghana Health Service (GHS) Field Epidemiology Training Programme which started in 2007 in the University of Ghana School of Public Health. Together with Kenya, Uganda and Zimbabwe FELTPs, these four Field Epidemiology Programme (FETPs) are the founding members of the Africa Field Epidemiology Network (AFENET). AFENET has since developed into a robust network of FE(L)TPs in over 33 countries in Africa. As the first FETP to be formed in West Africa [6], GFELTP has a vision of improving the health of the people in Ghana and beyond. The programme has a mission of contributing to addressing Ghana's public health needs and priorities through training and service provision in applied Epidemiology and Public Health Laboratory Management [7]. The GFELTP uses the One Health approach, ensuring teams of professionals from diverse backgrounds at the national and sub-national levels develop the capacity to early detect, investigate and respond to outbreaks in their districts. Guided by the World Health Organization International Health Regulations (WHO/IHR) core capacity requirements, the program strives towards building and strengthening national and local capacity to effectively respond to public health emergencies and mitigating their impact. GFELTP began with initial funding from the US CDC. The impact of the programme during its initial years proved that it was a great idea and could indeed strengthen the nation's public health systems. However, after five years, the funding from US CDC could not be continued. The programme had to identify other ways of running considering its contribution to solving public health issues in Ghana after its few years of existence. Sustainability has been one of the major challenges FETPs face [8]. One key aspect of sustainability is ensuring that programme services are maintained through the continuous provision of these services to the nation [8]. This can only be possible if sustainability strategies are in place. Sustainability of programmes has been found to go beyond just finances. Areas such as human resource, political support, operating environment and infrastructure have been considered as key determinants of sustainability [9]. A number of strategies in these major areas seem to have been adopted by GFELTP over time through the changing phases of the programme to enable its continuity. This paper assessed the sustainability strategies embedded in the Ghana Field Epidemiology and Laboratory Training Programme.

## Methods

**Study design**: the study was conducted in the GFELTP secretariat from March to June, 2017. A record review of database of residents and all programmatic documents was done. Interviews (key informant interviews (KII) were also carried out. We grouped the information collected under four main categories; programmatic, financial institutional and political structures. All components of the programme which contributed to ensuring continuous provision of the programme and its services were considered.

**Data collection**: data collection was done with a data extraction tool which covered information on residents' demographic characteristics, and available data on the indicators of sustainability we sought to assess. An interview guide based on the indicators of sustainability we sought to assess was used for the qualitative interviews. Questions on; how the programme has been funded since its inception, who the programme stakeholders were and the roles they played, how the programme runs and political structures governing the programme and the achievements of the programme since its inception were asked. We retrieved and extracted data from a database of residents enrolled onto the program from 2007 to 2017 and examined data on resident demographic information such as gender, nationality, and place of work before enrollment into the programme and their current places of work. All programme documents including the FELTP curricula, reports on residents, programme activities, and programme support files and administrative documents of the GFELTP from its inception were reviewed and information extracted. To understand the funding of the program, we reviewed the financial records and reports on funding activities and extracted needed information. We interviewed key informants such as the Foundation Director of the programme, Past Dean-School of Public Health, Director of Public Health-Ghana Health Service, CDC, WHO, Programme Directors past and present, Field Coordinator, Programme Coordinator and administrator. These interviews were done to give a more detailed understaning of the structures the programme has in place.

**Data processing and analysis**: we generated frequencies and proportions of residents' demographic characteristics. Recorded interviews were transcribed. We assessed sustainability by the following indicators: programme funding, programme evaluation, organisational capacity, institutional structures, political support, partnership and planning [9,10]. Documentation showing the presence and structures of the various indicators were extracted during the record review and clarity on their functionalities were triangulated by the interviews transcribed. In this paper, we defined donor, stakeholder and sustainability in the following ways. Donor: organisations which provide funds to solve specific health related challenges, in this case, those which provided funding to support the programme mainly with funds for a specific period [11,12]. Stakeholder: institutions which have a stake in the impact the program makes and partner to ensure the day-to-day smooth running of programme activities such as the Ministry of Health, Ghana Health Service, University of Ghana and AFENET [13,14]. Sustainability: the availability of human, financial, programmatic infrastructure and organizational resources to plan and provide services to meet needs and attain results on an ongoing basis to carry out core functions of the programme independent of individuals or one-time opportunities [2,15].

## Results

**Programme structure** *how the programme is run and its performance from 2007-2017*: GFELTP runs a two-tier training programme; frontline which is a three-month course and advanced which is a two-year training course which offers an MPhil degree. As of July 2017, the frontline course had trained seven cohorts and the advanced ten cohorts.

*Residents trained by GFELTP*: the programme has trained 350 people mainly from the Health Services with 94% (329) being Ghanaians. Residents of the programme were from all ten regions of Ghana (Table 1).

**Table 1:** Characteristics of residents enrolled in GFELTP, 2007-2016

	Trainees	
	Advanced (N=100)	Frontline (N= 250)
Characteristic of residents	n(%)	n(%)
Gender		
Male	79(79)	110(44)
Female	21(21)	140(54)
Nationality		
Ghanaian	87(87)	250(100)
Non-Ghanaian	13(13)	0
Track		
Epidemiology	38(38)	175(70)
Laboratory	46(46)	30(12)
Veterinary	16(16)	45(18)
Workplaces of residents at enrollment		
Ghana Health Service	65(65)	195(78)
Veterinary Service Directorate	16(16)	45(18)
Other institutions	7(7)	10(4)
International organizations	12(12)	0(0)
Number of regions in Ghana trainees worked in at time of enrollment (N=10)		
	Advanced	Frontline
Year	n(%)	n(%)
2007	3(30)	-
2008	6(60)	-
2009	3(30)	-
2010	5(50)	-
2011	7(70)	-
2012	6(60)	-
2013	7(70)	-
2014	5(50)	1(10)
2015	7(50)	3(30)
2016	7(70)	3(30)

*Performance of the programme*: overall, 100 outbreaks have been investigated. In all, 141 oral and poster presentations have been made by residents and alumni of the programme at national and international conferences. The presentations were made at AFENET and Training Programmes in Epidemiology and Public Health Interventions Network (TEPHINET) and other national and international conferences. A total of six awards were won during these conferences (Table 2).

**Table 2:** List of award winners and their topics

Conference/ venue	Award	Title
TEPHINET 2010, South Africa	Best Oral Presentation	Outbreak of Food Poisoning at a Salad Joint-Koforidua, Ghana, November 2009.
AFENET 2013, Ethiopia	2nd Runner Up for Oral Presentations	Obesity Associated Hypertension among a Religious Group in the Akwapim North District of Ghana, 2012
AFENET 2017, Abuja	1^st^ Runner up for Best Oral Presentation	Outbreak of Cholera in Vea-Gunga, Bongo District of the Upper East Region, Ghana
GFELTP 2017, Accra	Best Oral Presentation	Determinates of low birth weight in Neonates born in 3 hospitals in Brong Ahafo Region, Ghana- Unmatched case control study
GFELTP 2017, Accra	Best Poster presentation	Geospatial variation of confirmed malaria incidence and related environmental characteristics in sub-districts of Upper East Region
GFELTP 2017, Accra	1^st^ runner up for oral presentations	Prevalence and determinants of preterm delivery in an inner city referral hospital, Greater Accra Region, Ghana

*Positions occupied by Alumni of the programme*: Nearly 30% (20/70) of the alumni occupy positions as Directors of Public Health Service in the Ministry of Health, Ghana Health Service, and Veterinary Services Department. Twenty percent (14/70) also head various departments in the health facilities. However, 20% (14/70) have still not been placed by the Health Service after completion of the programme (Table 3).

**Table 3:** Positions occupied by alumni after completion of programme

Position	Frequency(N=70) n (%)
Director of health services	20 (28.6)
Disease control programmes	7 (10.0)
FELTP faculty	5 (7.1)
Heads of public health departments	14 (20.0)
Academia	10 (14.3)
Retained at positions before training	14 (20.0)

*Evaluation*: the GFELTP secretariat conducts monthly faculty meetings to evaluate the progress of the program. There were records of bi-annual evaluation of the programme, faculty and residents. These evaluations were conducted by residents. Likewise, the programme also evaluates residents enrolled in the programme. Though the programme is still in its preparatory phase of finalising a strategic plan, the AFENET strategic plan had been modified and adopted for their use. The programme was preparing to take up the TEPHINET accreditation when this review was done. WAHO also conducted a quality assurance evaluation of its partnership with the programme following a two-year collaboration. This process included review of documentation by the programme, interviewing of residents and faculty of the programme.

**Financial structure** *Funding of the programme*: the first four years of the programme was fully donor-funded by United States Centers for Disease Control and Prevention (CDC). The funding of the entire programme was taken over by stakeholders when funding ceased during the fifth year. Subsequently, funding from different donors over the years have been in the form of partial support for programme activities and assistance for residents training. The Dean of the School of Public Health supports some outbreak investigations with other projects overheads and residents' self-support. Other donors who have supported the programme include; The Presidential Malaria Initiative of the USAID which supports some residents annually in conducting malaria-based researches; US President's Emergency Plan for AIDS Relief (PEPFAR); and West Africa Health Organization (WAHO). The World Health Organization (WHO) also supported the training of frontline health workers among others. Overall, most programme activities have been funded with resources provided by stakeholders.

**Figure 1:**
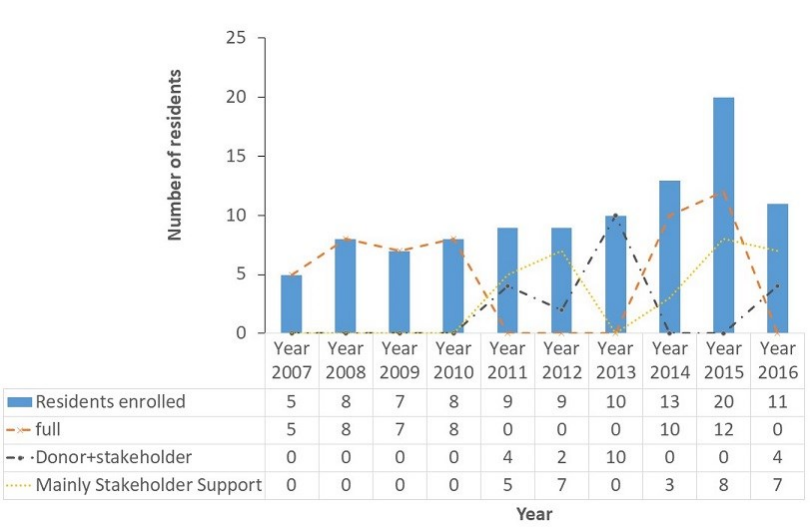
Source of tuition funding for residents enrolled onto the advanced course of the Ghana Field and Laboratory Training Programme, 2007-2016

*Funding of residents training*: over the years, the advanced course has been funded by donors, stakeholders and residents. Stakeholders played a key role in funding of residents over the years, providing support to half (50/100) of the residents. The following were some of the ways identified: residents who were mainly supported by stakeholders paid their tuition whiles the cost of field work, outbreak investigations and attendance of conferences was borne by stakeholders of the programme. The Dean of the School of Public Health also supported some outbreak investigations done by residents with overhead costs generated from other projects running in the school. Also, the Government of Ghana granted residents study leave during the training and paid their salaries throughout the FETP training (Figure 1). Self-support has been introduced for residents who do not have any additional support except for Government of Ghana Study leave with pay. They self-support their field work and tuition. The programme also offers service provision to other organisations as a source of revenue generation. However, the frontline FETP programme which has trained 200 frontline health workers in the country was fully funded by the WHO in 2015.

**Institutional structures of the programme** *Organizational capacity*: the programme is run by the School of Public Health, University of Ghana on behalf of the Ministry of Health/Ghana Health Service. The programme operates with the same principle used to operate the School of Public Health. As a university based programme, faculty of the university form the core faculty of the programme and are assigned to teach most of the courses. The University serves as a teaching site whiles the National/Regional/District Directorates and facilities of Ghana Health Services (GHS) and Veterinary Service Directorate (VSD) serves as field sites for the residency training. As a competency based programme training with the one health approach, various stakeholders provide technical expertise to teach the residents. Each year, guest lecturers from partner institutions such as CDC and WHO visit the program to deliver lectures.

*Stakeholders and Partnership with the programme*: the stakeholders of the programme include; Health Service (Ghana Health Services and Veterinary Services Department), University of Ghana, School of Public Health and AFENET. They play an intertwined role in providing resources (human, materials and funding). These stakeholders and partners collaborate in supporting the smooth running of all activities of the programme. The programme works with both local and international partners such as CDC Ghana Country Office, WHO Ghana Country Office, WAHO and Food and Drugs Authority (FDA), among others. These organisations collaborate with the programme in implementation of its activities. Stakeholders provide various resources in the form of human resource for field supervision, outbreak investigation, teaching and mentoring of residents and provision of financial resources for running of other programme activities.

*Faculty of the programme*: in addition to university staff on the programme, stakeholders also provide lectures in veterinary, laboratory and epidemiology in accordance with the One Health Approach. Periodically, alumni teach some didactic sessions of the courses.

*Alumni trained to become faculty*: for continuity and availability of staff, alumni are encouraged to pursue academia so that they can take up teaching appointments offered by the programme. A total of 4 graduates have completed their upgrade from MPhil to PhD with 10 currently enrolled unto PhD programmes. These alumni offer support to the programme and other programmes in other African countries such as Liberia, Sierra Leone and Namibia through teaching and mentorship of residents.

*Field supervision and mentorship of residents*: the programme has a strong alumni network which serves as a pool of experts for the programme. Supervision during field work/mentorship is done mainly by alumni at the districts, regional health directorate and national levels. Alumni work closely with the residents and supervise their acquisition of skills and knowledge, commonly referred to as bound volume competencies, with the assistance of Ghana Health Service staff. Some of the university faculty members periodically conduct field supervisory visits to the residents especially those from other West African countries such as Liberia and The Gambia.

**Political support**: the programme was established under the mandate of the Ministry of Health/Ghana Health Service and is engrained in the University of Ghana system. It therefore reports to the Director General of Ghana Health Service through the Director Public Health and to the Vice Chancellor of the University of Ghana through the Dean of School of Public Health. The reporting structures were agreed to through a memorandum of understanding signed between the two stakeholders. GFELTP has a steering committee that steers the affairs of the programme. The committee is chaired by Director of Public Health, Ghana Health Service. The steering committee is made up of stakeholders and partners. This committee meets at least twice a year to plan and take major decisions for the programme.

## Discussion

The GFELTP has been in existence since 2007. This paper sought to determine the sustainability structures embedded in the programme and other structures it has in place to ensure its smooth running. This assessment found programmatic, organizational, institutional and political sustainability strategies in the operation structures of GFELTP. The frontline training, introduced a few years ago as a means of reaching out to frontline health workers at district level; is a major sustainability strategy. This training provides frontline health workers with requisite skills to detect, monitor and manage outbreaks effectively [16]. It creates an avenue for publicising the programme and showcasing its good works at district and national levels. This visibility of GFELTP serves as a marketing strategy for the programme sustainability [8]. A strategy which has helped the sustainability of the programme was the fact that the residents were mainly staff of Ghana Health Services and the Veterinary Services Department. Residents trained were from all the ten regions of Ghana, demonstrating the wide coverage of the programme. After training, residents returned to serve in the ministry with the new competencies they have gained. The key to success of public health systems is availability of trained competent workforce. Therefore, as more health service staff are trained, the public health workforce which is critical to strengthening of the existing public health systems is reinforced [1]. GFELTP is the first port of call whenever an outbreak occurs in the country. Their experiences in handling outbreak investigations has shaped the process of investigating outbreaks in the country. There was evidence of the various outbreak reports documented by the residents. Most of these had been turned into abstracts which had been presented at local and international conferences. The periodic evaluations of programme activities by the GFELTP ensured the programme meets the required standard and gains recognition as a resource centre to help strengthen the public health system in the country. This has served as an opportunity to address gaps in the system and provide solutions for improvement. Sustainability has also been described by Schneider as a programmes ability to fund and manage itself with no external support [1]. The main source of funding of the programme was from stakeholders. Their support has been through contribution of materials, finances, human resource capacity and technical support. This has helped in saving the programme the costs for these essential services needed for the sustainability of the programme. The ability of stakeholders themselves to fund the activities of the programme is a sign of it being sustainable. Thus the GFELTP is on the track to achieving this goal of total independence and stability.

After support from the initial donors ceased, the programme reached out to other organisations to build partnerships to supplement stakeholder efforts in training residents. Survival of the programme during this time was of critical concern since sustaining grant-funded programmes after funding ceases has been known as a major public health challenge [16]. According to Adze and colleagues, exploring of other funding options as the programme did is essential in building collaborations with potential partners who can assist in developing the public health workforce capacity [8]. The programme expanded its collaboration with partners over the years and this has been of immense support to training of residents at frontline and advanced levels. Currently, this strategy is used by Central African FELTP [8]. Resource mobilization towards training of the residents was done by all stakeholders. The various stakeholders were found to have contributed in diverse ways to make the training of residents possible. Self-support has been introduced for residents who do not have any support from partners. These residents are allowed to use their own resources for some of the field work. The Programme has embarked on active service provision to other organizations that require its competencies as a means to generate additional resources for the sustainability of the programme. Considering the current existing structures of the programme, strong collaborations exist between programme stakeholders. A number of external partnerships are still being developed by the programme. Introduction of self-sponsorship and service provision (consultancies) to generate additional resources suggest that GFELTP is gradually building a stable and solid foundation in the country as a solution to developing a resilient public health response team. The GFELTP is a Ministry of Health/Ghana Health Service programme run by the University of Ghana like every other graduate programme. This provides the programme the opportunity to make use of university resources such as staff and infrastructure. This strategy was proposed and adopted in 2010 by the then dean of the school. The programme was therefore engrained in the university yet tied to a competence certification with over 70% field work. Thus, the programme can exist even in the face of little or very low financial support from donors. A university-run programme on behalf of the Ministry of Health/Ghana Health Service implied that individuals apply for admission through the university's regular system. Individuals also pay fees to the university just like every other course is run. In addition, faculty of the programme who are lecturers in the university have their salaries paid by the university and the staff from the Ghana Health Service paid by the Ghana Health Service. The core staff of the programme are therefore lecturers of the University of Ghana School of Public Health, the host institution and the rest from the Ministry of Health/Ghana Health Service. This strategy is similar to what is practiced in Chennai in India [17]. Having staff of the host institution as core faculty implies that there are key people who are always present to take major decisions on the programme as and when it is required. The variety of lecturers in the GFELTP exhibit the One Health Approach. This denotes the sign of a competently trained multidisciplinary public health workforce able to respond to health emergencies. This finding is confirmed by similar works done by others on FETP trainings in various parts of the world [3,18]. Incorporation of lecturers from different institutions leads to building of stronger ties between stakeholders, another sustainable approach to keep the programme running.

Should funds for the programme run out, the overhead cost of the programme are absorbed by the university through funds from other projects it runs. This has been one of the greatest sustainability strategies which has kept the programme running over the past years. Over the years, GFELTP has weathered the storm of financial challenges using this strategy and was saved from this major setback in its early years. This innovative idea adopted at the inception of the programme is in line with the principle that implementation and sustainability go hand in hand. Therefore, ways of ensuring the longevity of a programme need to be in place during the initial stages of implementation [19]. Resources and administrative support the programme needed were mainly provided by stakeholders who work collaboratively, performing various complementary roles and providing sufficient resources to support the programme. The programme collaborates with other organisations in order to carry out its activities successfully. Similar to other FELTPs in other parts of the world, these collaborators provide both technical and financial support for the programmes activities [20]. The strength of their partnership is seen in the makeup of the steering committee. The steering committee has representatives from all stakeholders of the programme on board. An assessment of in-service training in Swaziland revealed that sustainability of a training programme requires consulting and involving all stakeholders of the programme since this leads to the success of the programme [21]. We realized the sustainability plan was extended to recruiting staff for the programme who can take over from the first generation of leaders who began the programme. This was seen from the number of alumni in academia and those pursuing higher degrees after completion of the programme. Furthermore, alumni being called upon to assist in teaching residents provides an avenue for them to demonstrate what they have been taught and build their confidence in becoming future faculty of the programme. Supervision of residents by alumni and GHS staff have introduced residents to practical experiences in the various settings of the stakeholders. Also, this serves as an avenue for residents to share knowledge from didactic trainings with field staff. This approach to handling the various health challenges of the country is innovative [22]. Mentors are experienced epidemiologists who guide residents during their fieldwork. Mentors serve as role models ensuring that residents receive a well-rounded and complete training experience and competencies in the programme [1]. The programme places premium on mentorship thus alumni serve as mentors to guide residents and ensure they acquire the right skills and competences. The alumni network is a pool of resources the programme leverages for mentorship of the residents. This is in consonance with Subramanian and colleagues finding that FETP graduates are generally the first port of call for mentorship of the residents [20]. Selected graduates serving as mentors show their existing relationship with the programme after completion of training. This is a sustainability strategy using available human resource capacity of the programme. The programme has a strong political support because it is engrained in the university system and was established under the mandate of the Ministry of Health/GHS, the highest health delivery authority in the nation. The GHS chairs the GFELTP steering committee. The GHS being a key player in the management of the programme is a clear reflection of a solid sustainability strategy embedded in the programme. The programme was sustainable but had some challenges. The main programme challenges identified include; placement of residents after their training and gaps in programme funding. The programme needs to devise alternate ways of generating funds and other resources aside the traditional way of relying on stakeholders. The programme should invest more time into sourcing of projects from partners and writing of project proposals which would lead to diverse ways of funding the programmes activities.

**Limitations**: this review had a few challenges. Interviews were mainly recall of events that had happened in the past ten years. To reduce recall bias, different people were interviewed and the information obtained crosschecked for consistency with each other. Additionally, review of programme documents confirmed some of the interviews outcomes.

## Conclusion

The programme had evidence of stable sustainability strategies in all structures evaluated, namely; programme, financial, institutional and political. This assessment showed a strong collaboration between stakeholders who work hand in hand to ensure the programme runs smoothly. However, more sources of funding, broadening stakeholder partnership and other essential resources need to be considered to help the programme obtain a pool of resources for carrying out its activities.

### What is known about this topic

- The Ghana Field Epidemiology and Laboratory Training programme trains health workers to build a resilient public health workforce in Africa;
- Most Field Epidemiology and Laboratory Training programmes rely mainly on donor support to run their programmes and this could have implications on the programmes if the funds run out;
- Sustainability of Field Epidemiology and Laboratory Training programmes is essential and needs to be looked at by all programmes.

### What this study adds

- In the face of financial challenges due to the reduction in donor support, the Ghana Field Epidemiology and Laboratory Training programme was able to continue its mandate of training health workers;
- Aside donor support Field Epidemiology and Laboratory Training programmes can explore other alternatives of securing resources to run their programmes;
- The Ghana Field Epidemiology and Laboratory Training programme has innovative sustainability strategies integrated in its structure.

## Competing interests

The authors declare no competing interests.
